# Novel staphylococcal species that form part of a *Staphylococcus aureus*-related complex: the non-pigmented *Staphylococcus argenteus* sp. nov. and the non-human primate-associated *Staphylococcus schweitzeri* sp. nov.

**DOI:** 10.1099/ijs.0.062752-0

**Published:** 2015-01

**Authors:** Steven Y. C. Tong, Frieder Schaumburg, Matthew J. Ellington, Jukka Corander, Bruno Pichon, Fabian Leendertz, Stephen D. Bentley, Julian Parkhill, Deborah C. Holt, Georg Peters, Philip M. Giffard

**Affiliations:** 1 Menzies School of Health Research, Charles Darwin University, Darwin, Northern Territory, Australia; 2 Wellcome Trust Sanger Institute, Hinxton, Cambridge, UK; 3 Institute of Medical Microbiology, University Hospital Münster, Münster, Germany; 4 Public Health England, Cambridge, UK; 5 Department of Mathematics and Statistics, University of Helsinki, Helsinki, Finland; 6 Public Health England, London, UK; 7 Project Group Epidemiology of Highly Pathogenic Microorganisms, Robert Koch-Institut, Berlin, Germany

## Abstract

We define two novel species of the genus *Staphylococcus*that are phenotypically similar to and have near identical 16S rRNA gene sequences to *Staphylococcus aureus*. However, compared to *S. aureus* and each other, the two species, *Staphylococcus argenteus* sp. nov. (type strain MSHR1132^T^ = DSM 28299^T^ = SSI 89.005^T^) and *Staphylococcus schweitzeri* sp. nov. (type strain FSA084^T^ = DSM 28300^T^ = SSI 89.004^T^), demonstrate: 1) at a whole-genome level considerable phylogenetic distance, lack of admixture, average nucleotide identity <95 %, and inferred DNA–DNA hybridization <70 %; 2) different profiles as determined by MALDI-TOF MS; 3) a non-pigmented phenotype for *S. argenteus* sp. nov.; 4) *S. schweitzeri* sp. nov. is not detected by standard *nucA* PCR; 5) distinct peptidoglycan types compared to *S. aureus*; 6) a separate ecological niche for *S. schweitzeri* sp. nov.; and 7) a distinct clinical disease profile for *S. argenteus* sp. nov. compared to *S. aureus*.

There are over 40 species of the genus *Staphylococcus*, of which the coagulase-positive *Staphylococcus aureus* is a major cause of human clinical disease. The population structure of *S. aureus* is well-understood and comprises clonal complexes (CCs) with <2 % nucleotide divergence. Recently two *Staphylococcus*lineages have been recovered from human clinical infections (Holt *et al.*, 2011; Ng *et al.*, 2009; Ruimy *et al.*, 2010; Tong *et al.*, 2010), from non-human primates (Schaumburg *et al.*, 2012), and from bats in Africa (Akobi *et al.*, 2012). These lineages have previously been identified as *S. aureus *according to phenotype, but on the basis of multi-locus sequence typing (MLST) and a single genome sequence, the lineages are allied to but significantly diverged from *S. aureus*. We describe here investigations including the use of whole-genome sequence analysis to justify classification as three separate species, *S. aureus*, and two novel species of the genus *Staphylococcus*.

Strain MSHR1132^T^ was isolated from blood cultures of an Indigenous patient from Darwin, Australia (Holt *et al.*, 2011); and strain FSA084^T^ was isolated from the nares of a red-tailed monkey (*Cercopithecus ascanius*) from Gabon, Africa (Schaumburg *et al.*, 2012). Both strains grew on tryptone soy agar (TSA) at 37 °C with large, round, smooth colonies similar to typical *S. aureus*. Colonies of FSA084^T^ have a yellowish-pigmented appearance while those of MSHR1132^T^ are non-pigmented, displaying a creamy white appearance. The difference in pigmentation between typical *S. aureus* and strain MSHR1132^T^ is particularly evident after growing on chocolate agar (Oxoid) for 48 h at 37 °C (Holt *et al.*, 2011). Strains MSHR1132^T^ and FSA084^T^ are both catalase-positive, coagulase-positive by tube coagulase test and colonies demonstrate β-haemolysis on blood agar. Gram staining tests revealed Gram-stain-positive cocci in clusters for both strains. MLST revealed MSHR1132^T^ as ST1850 and FSA084^T^ as ST2022. We also selected for further investigation five strains that clustered according to MLST with MSHR1132^T^ (LBSA043, JABA32044, M260, M051, H115100079) and five that clustered with FSA084^T^ (FSCB1B, FSCB5, FSA096, FSA090, FSA037). The MSHR1132^T^ lineage strains were all recovered from human hosts from northern Australia (Brennan *et al.*, 2013; McDonald *et al.*, 2006), Fiji (Jenney *et al.*, 2014) and the UK. The FSA084^T^ lineage strains were recovered from non-human primates in Gabon and Côte d’Ivoire, Africa (Schaumburg *et al.*, 2012). These additional strains demonstrated the same cell and colonial morphology as MSHR1132^T^ and FSA084^T^ respectively. All MSHR1132^T^ lineage strains appeared non-pigmented.

Whole-genome sequencing using the Illumina HiSeq platform of the 12 strains, followed by core-genome single nucleotide polymorphism (SNP)-based maximum-likelihood trees demonstrated that MSHR1132^T^ lineage strains, FSA084^T^ lineage strains, and reference *S. aureus*genomes, form three distinct clusters with 100 % bootstrap support (Fig. 1). The details and GenBank accession numbers for these strains are provided in Table 1. Compared to *S. aureus*, the 16S rRNA gene sequence (1474 nt) is identical in strain MSHR1132^T^ and differs at one position in strain FSA084^T^. However, pairwise average nucleotide identity (ANI), as calculated using JSpecies (Richter & Rosselló-Móra, 2009), across the genomes within and between these groups was consistent with separate species designations (Table 2). Previously it has been demonstrated that an ANI <95 % corresponds well to a DNA–DNA hybridization (DDH) value of <70 % (Goris *et al.*, 2007). Similarly, an analysis using the Genome blast Distance Phylogeny (Meier-Kolthoff *et al.*, 2013) to calculate genome-to-genome distances clearly demonstrated three separate groups with mean inferred DDH values of 34 % and 36 % between *S. aureus* and the MSHR1132^T^ and FSA084^T^ lineages, respectively, 46 % between MSHR1132^T^ and FSA084^T^ lineages, and >80 % within lineages (Table 3). An analysis of orthologous core genes shared by all three groups using Bayesian Analysis of Population Structure (BAPS) software (Cheng *et al.*, 2013) demonstrated three BAPS clusters and an absence of admixture between the groups (Fig. 1). All MSHR1132^T^ lineage strains lacked the carotenoid pigment operon.

**Fig. 1.**
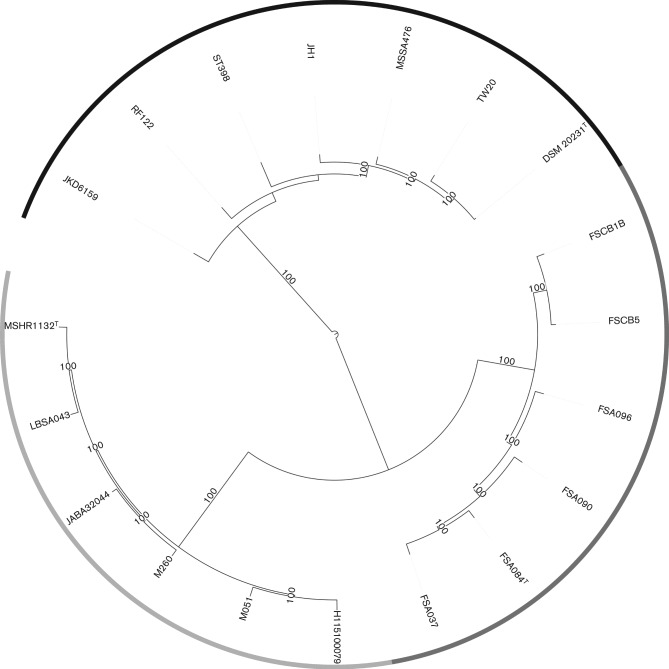
Maximum-likelihood phylogenetic tree of whole-genome sequence alignments for MSHR1132^T^ lineage strains (LBSA043, JABA32044, M260, M051, H115100079), FSA084^T^ lineage strains (FSCB1B, FSCB5, FSA096, FSA090, FSA037), and reference *S. aureus *strains (JKD6159, RF122, ST398, JH1, MSSA476, TW20, DSM 20231^T^). The outer circle shading indicates the three Bayesian Analysis of Population Structure (BAPS) groups: *S. aureus* (black), MSHR1132^T^ lineage (light grey) and FSA084^T^ lineage (dark grey). Branches with bootstrap support of 100 % are indicated.

**Table 1.** Strains and GenBank accession numbers for whole-genome sequences

Strain	Place of origin	GenBank accession nos of whole-genome sequences (for short-read and annotated assemblies, respectively)
*Staphylococcus argenteus* sp. nov.		
JABA32044V6S1	Fiji	ERS140248
CCEE01000001–CCEE01000018
LBSA043	Northern Australia	ERS140026
CCEM01000001–CCEM01000011
M051	Northern Australia	ERS140254
CCEN01000001–CCEN01000011
M260	Northern Australia	ERS140095
CCEF01000001–CCEF01000019
H115100079	UK	ERS154949
CCEP01000001–CCEP01000012
MSHR1132^T^	Northern Australia	FR821777
*Staphylococcus schweitzeri* sp. nov.		
FSA037	Gabon	ERS140147
CCEH01000001–CCEH01000058
FSA084^T^	Gabon	ERS140266
CCEL01000001–CCEL01000035
FSA090	Gabon	ERS140239
CCEO01000001–CCEO01000054
FSA096	Gabon	ERS140159
CCEK01000001–CCEK01000047
FSCB1B	Côte d’Ivoire	ERS140162
CCEG01000001–CCEG01000026
FSCB5	Côte d’Ivoire	ERS140167
CCEQ01000001–CCEQ01000038
*Staphylococcus aureus*		
DSM 20231^T^		AMYL01000000
JH1		CP000736
RF122		AJ938182
ST398		AM990992
MSSA476		BX571857
JKD6159		CP002114
TW20		FN433596

**Table 2.** Genome-wide average nucleotide identities (ANI) and inferred DNA–DNA hybridization (DDH) values for pairwise comparisons of strains from each of the three groups Values are mean with standard deviation. ANI was calculated using JSpecies (Goris *et al.*, 2007) and inferred DDH with Genome blast Distance Phylogeny (Meier-Kolthoff *et al.*, 2013).

Group		Pairwise comparison with:		
1	2	3
1. *S. argenteus* sp. nov.	ANI	98.8 (0.14)	92.0 (0.08)	87.4 (0.20)
DDH	89.1 (1.37)	46.4 (0.16)	33.5 (0.37)
2. *S. schweitzeri* sp. nov.	ANI		98.0 (0.44)	88.6 (0.14)
DDH		85.6 (4.32)	36.3 (0.23)
3. *S. aureus*	ANI			97.4 (0.49)
DDH			84.8 (8.00)

**Table 3.** Key biochemical tests used for identification of staphylococcal species Species: 1, *S. aureus* [number of strains (*n*) = 18]; 2, *S. schweitzeri* sp. nov. (*n* = 6); 3, *S. argenteus* sp. nov. (*n* = 6). Results were obtained in triplicate for each of MSHR1132^T^ lineage (*S. argenteus* sp. nov.) strains, FSA084^T^ lineage (*S. schweitzeri* sp. nov.) strains, and 18 ATCC strains of *S. aureus * using a Vitek2 GP Card (bioMérieux); see text for details of strains. Values are the proportion (%) of tests that were either positive or negative for each group of strains.

Biochemical test	1		2		3	
d-Xylose	–	(100)	–	(100)	–	(100)
Arginine dihydrolase 1	+	(100)	+	(100)	+	(100)
β-Galactosidase	–	(96)	–	(100)	–	(94)
Phosphatase	+	(100)	+	(100)	+	(94)
β-Glucuronidase	–	(100)	–	(100)	–	(100)
l-Pyrrolidonyl arylamidase	+	(100)	+	(94)	+	(94)
Urease	–	(100)	–	(100)	–	(56)
Polymixin B resistance	+	(94)	+	(100)	+	(100)
Lactose	–	(97)	–	(100)	–	(100)
*N*-Acetyl-d-glucosamine	+	(98)	–	(67)	–	(72)
Maltose	+	(100)	+	(100)	+	(100)
Novobiocin resistance	–	(98)	–	(67)	–	(100)
Growth in 6.5 % NaCl	+	(100)	+	(100)	+	(100)
d-Mannitol	+	(100)	+	(100)	+	(100)
d-Mannose	+	(100)	+	(100)	+	(94)
Raffinose	–	(100)	–	(100)	–	(100)
Sucrose	+	(100)	+	(100)	+	(100)
Trehalose	+	(83)	+	(100)	+	(100)
d-Ribose	–	(96)	+	(72)	–	(83)
d-Galactose	+	(82)	–	(56)	+	(100)

PCR amplification of the *nucA* gene that is used as a standard confirmatory marker for *S. aureus* is positive in strain MSHR1132^T^ but negative in strain FSA084^T^. An examination of the *nucA* gene and in particular the primer sites for *nucA* (Brakstad *et al.*, 1992) reveal one and two mismatches for the forward primer, and five and five mismatches for the reverse primer, for MSHR1132^T^ and FSA084^T^, respectively (Fig. 2). The presence of mismatches at the 3′ end of primers for strain FSA084^T^ most likely contributes to the lack of amplification of product for strain FSA084^T^. There were two in-frame deletions of 9 and 12 bp and one in-frame insertion of 3 bp in both MSHR1132^T^ and FSA084^T^ *nucA* sequences compared to *S. aureus*.

**Fig. 2.**
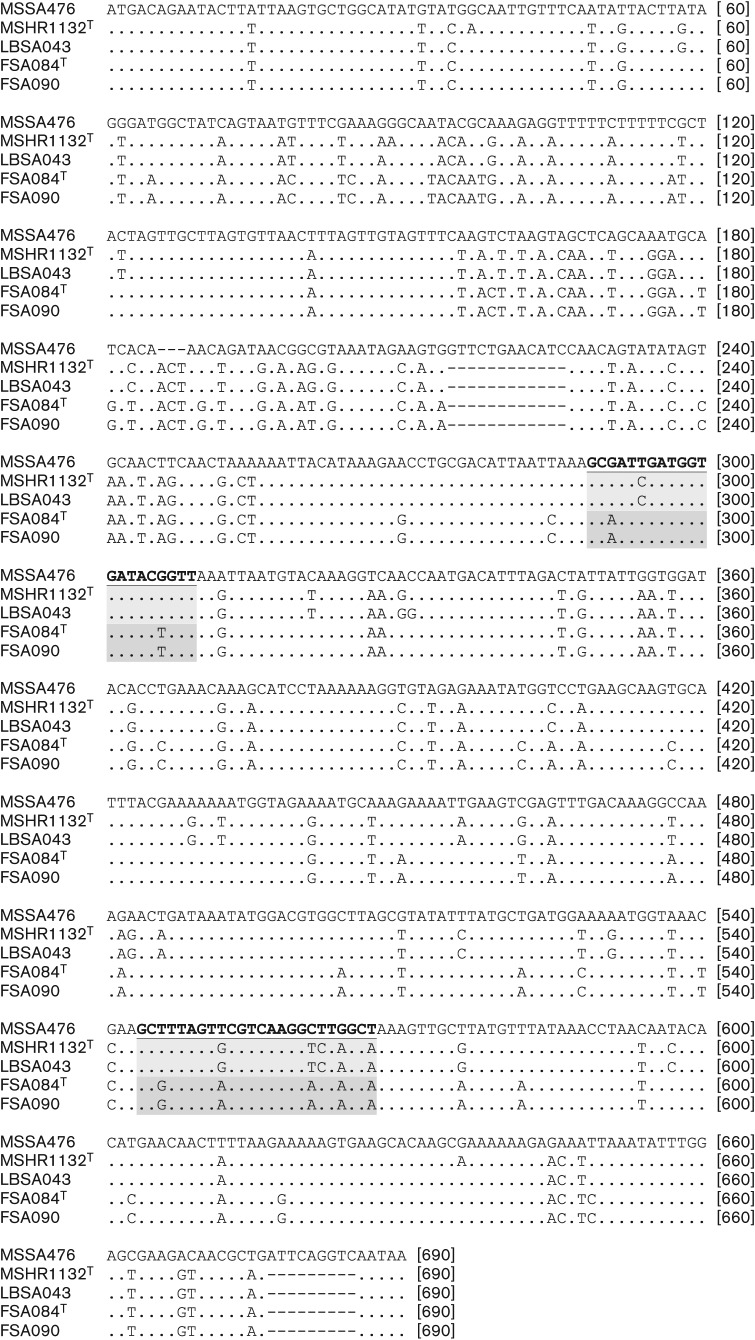
Sequence alignment of representative *nucA* gene sequences. The standard primer sites (Brakstad *et al.*, 1992) are indicated in bold underline (*S. aureus*), light grey shading (*S. argenteus* sp. nov.) or dark grey shading (*S. schweitzeri* sp. nov.). MSSA476 represents a reference *S. aureus*sequence, MSHR1132^T^ and LBSA043 represent *S. argenteus* sp. nov., and FSA084^T^ and FSA090 represent *S. schweitzeri* sp. nov.

Biochemical profiling was performed with the Vitek2 GP card platform (bioMérieux) according to the manufacturer’s instructions. We tested in triplicate each of the 12 strains together with 18 strains of *S. aureus* from the ATCC collection (ATCC 12600^T^, 13565, 13709, 14458, 19095, 19636, 23235, 25904, 25923, 27664, 29213, 29247, 33591, 33592, 43300, 49230, 49775, 51811) (Table 3). The biochemical test profiles for both MSHR1132^T^ lineage strains and FSA084^T^ lineage strains, are consistent with *S. aureus*, with mean probabilities of >95 % of identity as *S. aureus*. Although no test definitively discriminated between the three groups, the following may be helpful in identifying these lineages. The FSA084^T^ lineage strains were positive for d-ribose in 72 % of tests compared to 4 % and 17 % for *S. aureus* and MSHR1132^T^ lineage strains, respectively. The MSHR1132^T^ lineage strains were positive for urease in 56 % of tests compared to 0 % for both *S. aureus* and FSA084^T^ lineage strains. *S. aureus* was positive for *N*-acetyl-d-glucosamine in 98 % of tests compared to 33 % and 28 % for FSA084^T^ lineage strains and MSHR1132^T^ lineage strains, respectively.

We attempted to discriminate 12 MSHR1132^T^ lineage strains (an additional six strains to those already described), 12 FSA084^T^ lineage strains (an additional six strains to those already described), and 22 consecutive standard clinical strains of *S. aureus*by using matrix-assisted laser desorption ionization–time of flight mass spectrometry (MALDI-TOF MS) (Microflex LT MALDI-TOF instrument; Bruker Daltonik) (Table 4). We prepared samples using liquid phase formic acid extraction, according to the manufacturer’s recommendations, and compared the spectral profiles gained to the existing Bruker standard clinical database of profiles using MALDI Biotyper 2.1 software (Bruker Daltonik) with default settings. Strains of *S. aureus*were confidently identified. The MSHR1132^T^ lineage strains and FSA084^T^ lineage strains profiles were most similar to the *S. aureus*profile, but identity scores were much lower than for the strains of *S. aureus* (*P*<0.0001 for both compared to *S. aureus*) and fell below the manufacturer’s recommended threshold for a species level identification. We generated new reference profiles with three MSHR1132^T^ lineage strains and three FSA084^T^ lineage strains and repeated the analysis of all 46 strains. All strains were then confidently identified into their different groups. These findings are consistent with the three groups being separate species based on cell proteomic analysis.

**Table 4.** Comparison of MALDI-TOF MS identity scores using the standard clinical database and an amended database with reference profiles from *S. argenteus* sp. nov. and *S. schweitzeri* sp. nov. groups Identity score values are graded as highly probable species identification (score value 2.300–3.000), secure genus and probable species identification (2.000–2.299) and probable genus identification (1.700–1.999). Values are mean (standard deviation) of identity scores.

	Standard database		Amended database	
	Best hit	Identity score	Best hit	Identity score
*S. aureus* (*n* = 22)	*S. aureus*	2.295 (0.067)	*S. aureus*	2.295 (0.067)
*S. argenteus* sp. nov. (*n* = 12)	*S. aureus*	2.071 (0.102)	*S. argenteus* sp. nov.	2.700 (0.066)
*S. schweitzeri* sp. nov. (*n* = 12)	*S. aureus*	1.847 (0.095)	*S. schweitzeri* sp. nov.	2.676 (0.072)

Analyses of fatty acids, respiratory quinones and peptidoglycans were carried out by the Identification Service of the Leibniz-Institut DSMZ (Deutsche Sammlung von Mikroorganismen und Zellkulturen), Braunschweig, Germany. *S. aureus* ATCC 29213, MSHR1132^T^ and FSA084^T^ were cultured and tested for fatty acid composition under identical conditions. The fatty acid profiles of strains MSHR1132^T^ and FSA084^T^ were similar and dominated by anteiso-C_15 : 0_ and anteiso-C_17 : 0_ and corresponded in their composition to *S. aureus* ATCC 29213 (Table 5). Strains MSHR1132^T^ and FSA084^T^ contained the menaquinones MK-7, MK-8 and MK-9 at ratios of 11 : 70 : 11 plus a non-identified peak (MSHR1132^T^) and 7 : 80 : 13 (FSA084^T^). Menaquinones with seven to eight side-chains are characteristic for the genus *Staphylococcus* (Gotz *et al.*, 2006). Both MSHR1132^T^ and FSA084^T^ showed the same peptidoglycan type (A3α type, A11.8 type, l-Lys–l-Ala–(Gly)_4-5_) (Schleifer & Kandler, 1972; Schumann, 2011) (http://www.dsmz.de/?id=449). This peptidoglycan type is reported from *Staphylococcus vitulinus* (Švec *et al.*, 2004; Zakrzewska-Czerwińska *et al.*, 1995), *Staphylococcus lentus* (Schleifer *et al.*, 1983) and *Staphylococcus sciuri* (Kloos *et al.*, 1976), and is distinct from that of *S. aureus* (A3α type, A11.2 type, l-Lys–(Gly)_4-5_) (Gotz *et al.*, 2006).

**Table 5.** Cellular fatty acid contents of *Staphylococcus aureus**, Staphylococcus argenteus* sp. nov. and *Staphylococcus schweitzeri* sp. nov. Strain: 1, *S. aureus* ATCC 29213; 2, *S. argenteus* sp. nov. MSHR1132^T^; 3, *S. schweitzeri* sp. nov. FSA084^T^. Values are percentages of total fatty acids.

Fatty acid	1	2	3
C_14 : 0_	0.3	0.2	0.2
C_16 : 0_	2.1	2.1	1.9
C_17 : 0_	0.4	0.4	0.3
C_18 : 0_	6.3	5.8	6.0
C_18 : 1_ω9*c*	2.0	2.4	2.1
C_18 : 1_ω7*c*	0.2	0.0	0.2
C_18 : 2_ω6,9*c*/anteiso-C_18 : 0_*	0.8	0.9	0.7
C_19 : 0_	0.5	0.4	0.3
C_20 : 0_	2.6	1.6	1.7
C_20 : 1_ω9*c*	0.4	0.9	0.6
C_20 : 2_ω6,9*c*	0.2	0.0	0.3
iso-C_13 : 0_	0.1	0.0	0.0
iso-C_14 : 0_	0.4	0.5	0.6
iso-C_15 : 0_	5.4	7.2	5.8
iso-C_16 : 0_	0.8	1.1	1.3
iso-C_17 : 0_	3.1	4.6	4.0
iso-C_18 : 0_	0.3	0.4	0.5
iso-C_19 : 0_	0.9	1.0	1.0
anteiso-C_13 : 0_	0.1	0.0	0.1
anteiso-C_15 : 0_	50.2	48.5	47.1
anteiso-C_17 : 0_	20.4	19.6	22.3
anteiso-C_19 : 0_	2.9	2.4	3.2
Total	100	100	100

* Differentiation between these two fatty acids was not possible.

In Conclusion, although MSHR1132^T^ lineage strains and FSA084^T^ lineage strains share identical or near identical 16S rRNA gene sequences, have similar fatty acid and menaquinone compositions to *S. aureus*, and are phylogenetically the closest known relatives of *S. aureus*, there are strong justifications for assigning these lineages to two novel species of the genus *Staphylococcus*, for which the names *Staphylococcus argenteus* sp. nov. (type strain MSHR1132^T^) and *Staphylococcus schweitzeri* sp. nov. (type strain FSA084^T^) are proposed. These justifications are: 1) phylogenetic distance, lack of admixture, ANI <95 %, and inferred DDH <70 %; 2) different profiles as determined by MALDI-TOF MS; 3) non-pigmented phenotype of *S. argenteus* sp. nov.; 4) *S. schweitzeri* sp. nov. cannot be detected by standard *nucA* PCR; 5) distinct peptidoglycan types compared to *S. aureus*; 6) a separate ecological niche for *S. schweitzeri* sp. nov., which has only once been recovered from human hosts to date (Schaumburg *et al.*, 2012); 7) distinct clinical disease profile for *S. argenteus* sp. nov. compared to *S. aureus* (Tong *et al.*, 2013).

## Description of *Staphylococccus argenteus* sp. nov.

*Staphylococcus argenteus* (ar.gen′te.us. L. masc. adj. *argenteus* silver, silvery).

Colonies are large, 2 mm in diameter, round, convex, smooth, creamy white and demonstrate β-haemolysis on blood agar. The difference in pigmentation between typical *S. aureus* and *S. argenteus* is particularly evident after growing on chocolate agar for 48 h at 37 °C. Cells are Gram-stain-positive, coccoid, 1 µm in diameter, and form clusters. Facultatively anaerobic. Cells are catalase-positive and coagulase-positive by tube coagulase test. Biochemically positive for alkaline phosphatase, arginine dihydrolase, l-pyrrolidonyl arylamidase, galactose, maltose, mannitol, mannose, methyl β-d-glucopyranoside, sucrose, and trehalose; and negative for urease, α-glucosidase, phosphatidylinositol phospholipase C, β-galactosidase, alanine-phenylalanine-proline arylamidase, l-aspartic acid arylamidase, α-mannosidase, β-glucuronidase, l-leucine arylamidase, proline arylamidase, α-galactosidase, alanine arylamidase, tyrosine arylamidase, amygdalin, xylose, α-cyclodextrin, sorbitol, ribose, lactose, *N*-acetylglucosamine, pullulan, raffinose, salicin (Vitek2 GP Card). The peptidoglycan is of the type A3α, A11.8, l-Lys–l-Ala–(Gly)_4-5_. The menaquinones MK-7, MK-8 and MK-9 are at ratios of 11 : 70 : 11 and the predominant fatty acids are anteiso-C_15 : 0_ and anteiso-C_17 : 0_.

The type strain MSHR1132^T^ ( = DSM 28299^T^ = SSI 89.005^T^) was isolated from the blood culture of a 55-year-old Indigenous Australian female in 2006 in Darwin, Northern Territory, Australia. The type strain has also been deposited in the Robert Koch Institute (Germany) and the National Collection of type Cultures, Public Health England (UK).

## Description of *Staphylococcus schweitzeri* sp. nov.

*Staphylococcus schweitzeri* (schwei′tzer.i. N.L. gen. n. *schweitzeri* of Schweitzer, named after Albert Schweitzer, founder of a hospital in Lambaréné, Gabon, and Nobel Peace Prize Laureate in 1952).

Colonies are round, 1.7 mm in diameter, convex, smooth, yellow and demonstrate β-haemolysis on blood agar. Cells are Gram-stain-positive, coccoid, 1 µm in diameter, and form clusters. Facultatively anaerobic. Cells are catalase-positive and coagulase-positive by tube coagulase test. Biochemically positive for alkaline phosphatase, arginine dihydrolase, l-pyroglutamic acid arylamidase, maltose, mannitol, mannose, methyl β-d-glucopyranoside, sucrose, trehalose, ribose and *N*-acetylglucosamine, but negative for phosphatidylinositol phospholipase C, α-glucosidase, β-galactosidase, urease, alanine-phenylalanine-proline arylamidase, l-aspartic acid arylamidase, α-mannosidase, β-glucuronidase, l-leucine arylamidase, proline arylamidase, α-galactosidase, alanine arylamidase, tyrosine arylamidase, amygdalin, xylose, α-cyclodextrin, sorbitol, galactose, lactose, pullulan, raffinose and salicin (Vitek2 GP Card; bioMérieux). The peptidoglycan is of the type A3α, A11.8, l-Lys–l-Ala–(Gly)_4-5_. The menaquinones MK-7, MK-8 and MK-9 are at ratios of 7 : 80 : 13 and the predominant fatty acids are anteiso-C_15 : 0_ and anteiso-C_17 : 0_.

The type strain FSA084^T^ ( = DSM 28300^T^ = SSI 89.004^T^) was isolated from the nares of a non-human primate (*Cercopithecus ascanius*) from Gabon, Africa within 12 h after the death of the animal in 2010. The type strain has also been deposited in the Robert Koch Institute (Germany) and the National Collection of type Cultures, Public Health England (UK).
